# The Indirect Relationship Between Religious Practices and Egoism at Work Through Dark-Triad Traits? A Sample of Polish Employees

**DOI:** 10.1007/s10943-025-02363-x

**Published:** 2025-07-03

**Authors:** Marcin Wnuk

**Affiliations:** Department of Work and Organizational Psychology, Adam Mickiewicz University, ul. Szamarzewskiego 89/AB, 60–568 Poznań, Poland

**Keywords:** Religious practices, Relationship with god, Dark triad, Narcissism, Machiavellianism, Psychopathy, Instrumental ethical climate, Egoism at work

## Abstract

Religious involvement promotes employee virtues and encourages prosocial behavior in the workplace. There is no research about the preventive role of religion regarding dark-triad personality traits as antecedents of antisocial behavior at work. This study verified the mechanisms underpinning the relationship between religious practices and egoism at work, focusing on the mediating role of the dark triad and the moderating roles of perception of a relationship with God and an instrumental ethical climate. It was hypothesized that among Polish employees, according to relational spirituality, religious practices have a negative effect on dark-triad personality traits only in group with the most positive perception of a bond with God. It was also hypothesized that, consistent with the concept of trait activation, an instrumental ethical climate in turn strengthens the positive effect of dark-triad personality traits on egoism at work. In the cross-sectional study participated 434 employees from Poland. Consistent with the relational spirituality approach and concept of trait activation, the preventive role of the interactive effect of prayer and perception of a relationship with God for dark triad was confirmed the same as the moderating function of an instrumental ethical climate in the relationship between psychopathy and egoism at work and Machiavellianism and egoism at work.

## Introduction

According to data from 2024, 78% of Poles declared themselves believers (Bożewicz, 2024). Religious values are a major factor that influences national identity (Osewska et al., 2021). For 64% of Poles, being a Christian is very important to their national identity (Pew Research Center, 2018). Religious values and religious engagement can have an impact on the workplace, but this topic has not been thoroughly investigated yet. Recent research has shown that the role of religion in the workplace is still a neglected area of study that needs further exploration (Gundolf & Filter, 2012; Obregon et al., 2022), and the ethical and spiritual motivations of employees are still overlooked (Guillen et al., 2015). The vast majority of studies on this topic have been conducted in the USA. In Obregon et al.’s review of 52 papers published between 1960 and 2018 that refer to religiosity, spirituality, and work in their analysis, only three were conducted in European countries.

Some authors claim that religion is not a worthy subject for consideration in the workplace context (Mitroff & Denton, 1999), and others refer to the absence of evidence of the impact of this phenomenon on business ethics (Jurkiewicz & Giacalone, 2004). Additionally, in the literature, there is an observable trend toward the popularization of spirituality and the rejection of religiosity—spirituality is ascribed a positive meaning and religiosity a negative one. Spirituality is perceived as a subjective, individual, internal, dynamic, and positive process involving personal feelings, experiences, and thoughts, in contrast to objective, institutional, external, and static religion, connected with rituals, worship, and practices (Baumsteiger & Chenneville, 2015; Pargament, 1999). This approach has also appeared in the area of business ethics, where spirituality is connected with inclusiveness, flexibility, and tolerance, and religion with dogma, rigidity, and exclusiveness (Klenke, 2005; Mitroff, 2003). Previously, the majority of studies have focused on the positive outcomes of religion in the workplace, emphasizing its role in promoting moral virtues such as gratitude (Hardy et al., 2014; Naseer et al., 2020; Wnuk, 2018), empathy (Naseer et al., 2020; Hardy et al., 2014), forgiveness (Davis et al., 2010; Hardy et al., 2014; Wnuk, 2022a), and humility (Wnuk, 2024), rather than preventing negative ones like narcissism, psychopathy, and Machiavellianism.

Furthermore, religion has been considered a facilitator of positive attitudinal qualities in the workplace, like an ability to find meaning in work (David & Iliescu, 2022), organizational commitment (Kutcher et al., 2010), and job satisfaction (Kutcher et al., 2010) as well as prosocial behaviors like altruism (Lee et al., 2022), change-oriented citizenship behavior (Haq et al., 2018), and less acceptance of questionable ethical behaviors (Conroy & Emerson, 2004; Longenecker et al., 2004).

There is a lack of research on how religiosity prevents manifestations of negative personality traits that transfer into antisocial and unethical behavior at work. This study aims to fill the gap in this research by presenting and verifying the mechanisms underpinning the link between religious practices and egoism at work.

It is essential to call to the role of priest and nun, where religious involvement is an important element of daily functioning and requires an altruistic attitude, humility, gentleness, and lack of impulsivity. It is also significant in social jobs where empathy, good emotional regulation, and lack of cynicism are crucial, such as among psychologists, therapists, social workers, medics, or nurses. Earlier studies focused on the positive function of religiosity in the workplace without explaining the mechanism of this influence. They reached the limited conclusion that intrinsic religious motivation in the workplace leads to benefits, but extrinsic religious motivation is not related to any outcomes (Błażek & Besta, 2012; Vitell et al., 2009) or has detrimental consequences (Vitell et al., 2009).

### Religiosity

According to Koenig et al. (2001, p. 18), religion is “an organized system of beliefs, practices, rituals, and symbols designed (a) to facilitate closeness to the sacred or transcendent (God, higher power, or ultimate truth/reality), and (b) to foster an understanding of one’s relations and responsibility to others in living together in a community”. Following Saroglou (2011), religiosity is a multifaceted construct, which can be captured by four dimensions: believing, behaving, bonding, and belonging. Believing is realized through religious orthodoxy; behaving, through religious practices; bonding, by vertical faith maturity and spiritual struggles; and belonging, through religious identity.

The bonding aspect of religiosity is the most important element of relational spirituality, reflecting the relevance of attachment to God and His image in spiritual growth (Davis et al., 2021). The two most significant religious practices in the process of developing a bond with God are prayer, as an individual form of religious involvement, and Mass attendance, as a public manifestation of worship. According to James (1902/1958), prayer is a form of communication or conversation with God or another higher power perceived as divine. In the Polish population, 27% pray daily, and 61% attend religious services at least monthly (Pew Research Center, 2018). The popularity of praying is connected to its inherent availability and the lack of associated restrictions around place and time because this practice can be performed at any time and in any place, even the workplace. The top ranking of Poles in Europe regarding the frequency of Church attendance can be explained by historical and cultural factors, especially the significant role of the Roman Catholic Church in shaping national identity (Osewska et al., 2021; Pew Research Center, 2018).

The freedom to choose the place and time of prayer goes hand in hand with the free will to choose the type of praying appropriate to one’s needs: meditative, ritualistic, petitionary, colloquial (Paloma, & Pendleton, 1989; Paloma, & Pendleton, 1991), adoration, thanksgiving, reception, confession, or supplication (Laird et al., 2009).

### The Dark Triad

The dark triad is a term coined by Paulhus and Williams (2002) that recognizes some similarities between three personality traits: narcissism, Machiavellianism, and psychopathy. This three-factor construct has a long history in personality psychology. Chronologically, the first trait identified was Machiavellianism, by Christie and Geis (1970) in their book on the subject. Nine years later, Raskin and Hall (1979) presented a paper on their narcissistic personality inventory, and a year after that, Hare (1980) proposed criteria to diagnose psychopathy. Despite the fact that they have common attributes that overlap, such as manipulativeness, self-assuredness, self-centeredness, arrogance, and callousness (Kay & Arrow, 2022), they are distinguished by other unique, specific characteristics.

The results of previous studies indicated that dark-triad traits are the consequence of nurture (Jonason et al., 2014; Yendell et al., 2022). Jonason et al. (2014) showed that the quality of parental care is related to attachment patterns, which, in turn, are antecedents of different facets of the dark triad. For example, the mother's quality of parental care predicts a fearful attachment pattern, which is related to Machiavellianism.

Psychopathy consists of two factors (Hare et al., 1990), and each of them has two facets (Hare & Neumann, 2005). The first one involves the interpersonal aspect of manipulativeness and deceit and the affective facet—characterized by an inability to accept responsibility for one’s actions and a lack of empathy and remorse. The second factor is manifested by lifestyle issues, which involve irresponsibility and impulsivity, and antisociality, characterized by poor behavioral control and criminal versatility.

Christie and Geis (1970), in developing the construct of Machiavellianism, were inspired by the character described in the book *The Prince* (Machiavelli, 1532/2006), authored by Italian diplomat Niccolò Machiavelli (1469–1527). Machiavellianism refers to a mix of duplicity and cunning that is expressed by the attitude that “the ends justify the means.” This personality trait is characterized by a lack of empathy, exploitive treatment of other people to achieve one’s own aims, a cynical perspective on human nature as weak and untrustworthy, an absence of affect, and a lack of concern for others, a moral compass, and any ideology except that of driving to achieve self-interested purposes.

Du et al. (2021) examined the Five Factor Machiavellianism Inventory and combined it with measures of psychopathy and narcissism. They established a shorter version of the measures regarding Machiavellianism, distinct from psychopathy and narcissism, which includes 13 subscales, each of them part of three factors: antagonism, agency, and planfulness. Antagonism is reflected in selfishness, immodesty, manipulativeness, callousness, and cynicism; agency in being achievement-oriented, active, assertive, competent, self-confident, and invulnerable; and planfulness in acting in a deliberate and ordered manner.

Generally, narcissism entails an excessive focus on the self, leading to manipulativeness, callousness, grandiosity, attention-seeking, risk-taking, deceitfulness, and suspiciousness (Miller et al., 2022). This character trait has two types, which have different nomenclature in the literature but are most frequently labeled as overt and covert narcissism (Akhtar & Thomson, 1982) or grandiose and vulnerable narcissism (Koepernik et al., 2022). This first one involves assertiveness, gregariousness, an achievement orientation, and the propensity to fantasize, but the second one is based on vulnerability, self-consciousness, and the tendency to feel shame (Miller et al., 2022). Recently, they have been treated as two manifestations of narcissism within the trifurcated model of narcissism, which in addition to them, contains an antagonistic aspect of narcissism characterized by angriness, callousness, immodesty, distrustfulness, exploitation, and excitement seeking (Miller et al., 2022). The trifurcated model is theoretically well-grounded and empirically sound (Schneider et al., 2023).

### Religiosity and the Dark Triad

As mentioned earlier, previous studies have emphasized the role of religion in promoting virtues at work, neglecting the function of religious involvement in preventing of the expression detrimental attitudes in the workplace that are a result of dark-triad personality traits.

Among the dark-triad personality traits, the most frequently considered in the religious context has been narcissism, but the results of such research have been inconsistent and dependent on the type of narcissism, religiosity operationalization, and study sample, which were taken into account. For example, in a study of Italian priests, religious brothers, and religious sisters, intrinsic religiosity was unrelated to narcissism (Francis & Crea, 2021). In a US adult sample, perceived sacredness in life was negatively correlated with narcissism both for theistic and non-theistic participants (Doehring et al., 2009). Different results were achieved in Hermann and Fuller's study (2017), where non-religious US individuals had lower overall levels of narcissism than religious and spiritual but non-religious individuals. Unexpectedly, in the religious group, church attendance was not related to narcissism, but it was positively related to this personality trait among spiritual but non-religious individuals. In other studies, covert and overt narcissism were examined, and it was shown that religiosity plays another function for individuals with these two different types of narcissism. Overt narcissism is characterized by feeling influence and power over others and having a grandiose image of oneself; it is reflected in meditative prayer, which is directed at the self without the need for God’s presence or the desire for His support. Covert narcissism manifests a vulnerability to being hurt by the opinions of others and leads to idealizing God in contrast to one’s own weakness, which can be compensated for by a relationship with God and belief in His protection (Zondag & Uden, 2014). These two kinds of narcissism can be reflected in an individual’s type of religious beliefs, religious feelings, behaviors, and coping. Zondag and Uden (2010) found that covert narcissism was positively correlated with frequency of prayer and self-directing religious coping and negatively correlated with collaborative and deferential religious coping, but overt narcissism was not related to these religious indicators.

Little is known about the relationship between religiosity and both Machiavellianism and psychopathy. Intuitively, it can be assumed that if Judeo-Christian axiology is focused on love and service to other people, it will be negatively related to Machiavellianism and psychopathy as phenomena rooted in opposite traits and values. The results of recent research have confirmed that different aspects of religiosity, like intrinsic religiosity, workplace spirituality, and spiritual leadership, are negatively correlated with Machiavellianism (Francis & Crea, 2021; Zou et al., 2023). For example, in a sample of students from Indonesia, highly religious consumers had statistically lower levels of Machiavellianism in comparison with two other groups (Arli et al., 2019).

Contrary to expectations, among Muslim students, both behavioral and affective measures of religiosity were not related to levels of psychopathy (Fekih-Romdhane et al., 2020). Taking into account that the religious denomination and national level of religiosity can be moderators in the relationship between religiosity and dark-triad personality traits—and that what aspect of religiosity is considered in research can be a factor—in Roman Catholic employees from Poland, a negative correlation between religious practices and psychopathy was expected, as with the other two dark personality traits.

H1: In a sample of Polish employees, religious practices will be negatively correlated with dark-triad personality traits.

### A Perception of a Bond with God as a Moderator in the Relationship between Religious Practices and the Dark Triad

Consistent with the relational spirituality approach, religious practices become effective and lead to beneficial effects under some conditions, like convincement about God’s responsiveness and His engagement based on an internal representation of a relationship with God (Exline et al., 2021). The perception of a relationship with God may be filled with negative images of God, such as God as a punisher, a revenger, an abandoner, or inert. Or, God can be perceived as exhibiting positive qualities like forgiveness, love, support, mercy, openness, sensitivity, and being a “secure base” in times of crisis, threat, and stress at work. Religious practices are good opportunities to develop a bond with God. For example, in a sample of Polish employees’ perception of the relationship with God as a source of support, coping, and transcendence, moderated the link between prayer and both humility (Wnuk, 2024). Only in a group of individuals with higher-than-average results in an assessment of their relationship with God prayer was positively related to humility.

Similar outcomes were noticed in other studies, where an insecure attachment to God negatively predicted humility (Jankowski & Sandage, 2014) or was a premise for developing this virtue (Jankowski et al., 2022).

H2: In a sample of Polish employees, a relationship with God will moderate the relationship between religious practices and dark-triad personality traits.

### Religiosity and Unethical and Antisocial Attitudes: The Moderating Role of a Perception of a Relationship with God

Although some authors are opponents of researching religiosity in the workplace (Mitroff & Denton, 1999) and express skepticism regarding the influence of it on business ethics (Jurkiewicz & Giacalone, 2004), some studies have been conducted in this area and have confirmed religious involvement as an antecedent of work attitude.

In explaining the mechanisms underlying the relationship between religiosity and moral intentions, emotions, and behaviors, some moderators have been considered, such as religious orientation (Vitell et al., 2009), quality of spiritual experience (Hardy et al., 2014), organizational adversity with respect to voluntarism (Haq et al., 2018), and image of God (Greenway et al., 2018). For example, in Vitell et al.’s study (2009), religious orientation moderated the link between religiosity and moral identity internalization in a way that intrinsic religiosity correlated positively with moral identity internalization, but extrinsic religiosity was negatively related to moral identity internalization. Hardy et al. (2014) found that the quality of spiritual experience strengthens the link between daily spiritual experience and moral emotions such as empathy, gratitude, and forgiveness.

Among Christians from the USA, their image of God was verified as a potential moderator linking prayer and prosocial behaviors (Greenway et al., 2018). The effect of intercessory prayer on monetary donations was moderated by the traditional concept of God. The lower the score the participants received for their belief in the traditional concept of God, the stronger the effect of prayer on monetary donations was. Inversely, prayer correlated with lower monetary donations among those possessing the more traditional concept of God.

Among Pakistan employees, organizational adversity concerning voluntarism was a moderator in the association between religiosity and change-oriented citizenship. In this study, organizational adversity with respect to voluntarism strengthened the effect of religiosity on change-oriented citizenship behavior (Haq et al., 2018). Also, in a sample of US Christians, the moderating role of the image of God in reference to the connection between prayer and prosocial behaviors was noticed (Greenway et al., 2018). A more traditional concept of God weakened the positive relationship between prayer and monetary donations.

Egoism is one of the most important antisocial qualities and a significant factor in business ethics and employee well-being (Newman et al., 2017; Parboteeah et al., 2024; Weber & Opoku-Dakwa, 2022) that is not well-recognized in the workplace context (Wnuk, 2023). One of the reasons for this state of affairs is the lack of measures examining egoistic motivation at work and the tendency of researchers to focus on prosocial behaviors like altruism (Wnuk, 2023).

Egoism at work is an attitude toward one’s work, employer, and co-workers. It consists of caring only for one’s interest, an instrumental treatment of principles, rules, and values by observing them only when it gives tangible benefits, and the belief that one’s interest does not go hand in hand with the interests of other employees, which, at best, leads to indifference to their needs, desires, and aspirations. It assumes that the employee undertakes only actions that favor his well-being, without the possibility of actions aimed at the good of others, such as any altruistic or helpful and supportive behavior (Wnuk, 2023).

The vast majority of the studies considered egoism in the workplace from the organizational, not individual, perspective, indicating a harmful effect of instrumental ethical climate, which encourages employees to take care of their interests, ambitions, values, and needs, even the cost of workmates (Newman et al., 2017; Parboteeah et al., 2024).

Victor and Cullen's (1988) approach to organizational climate distinguishes five types of ethical climates: caring, law and codes, rules, instrumental, and independence. These types were differentiated based on three levels of the ethical dimension of decisions (individual, local, cosmopolitan). This study examined the instrumental ethical organizational climate, which focuses on employees’ egoistic motives and encourages them toward egoistic behavior and achieving self-interested goals. Employees who perceive the instrumental climate at work are more prone to moral disengagement (Pagliaro et al., 2018), turnover intention (Joe et al., 2018), assess the organization as injustice (Tziner et al., 2015), have a problem with organizational identification (Barattucci et al., 2021; Pagliaro et al., 2018), frequently present dysfunctional behavior (Parboteeah et al., 2024), and are less committed (Parboteeah et al., 2024; Tsai & Huang, 2008). For example, Barattucci et al. (2021) found that the perceived friendship ethical climate, through organizational identification, is indirectly positively related to organizational commitment, organizational citizenship behaviors, organizational trust, and organizational recommendation. In their study, the perception of individual ethical climate was not correlated with organizational identification and negatively predicted organizational trust. Also, Pagliaro et al. (2018) showed the negative indirect effects of self-interest ethical climate on organizational citizenship behaviors through moral disengagement and problems with organizational identification and the positive indirect effects of perception of this type of climate on counterproductive work behaviors.

Only one recent study has explored prayer and egoism at work connection on the individual level, showing that employees' egoistic motivations in the workplace are explained by the interactive effect of prayer and perception of a relationship with God (Wnuk, 2024). A relationship with God strengthened the negative link between prayer and an egoistic attitude in the workplace.

H3: In a sample of Polish employees, religious practices will be negatively correlated with an egoistic attitude at work.

H4: In a sample of Polish employees, a relationship with God will moderate the perception of a relationship between religious practices and an egoistic attitude at work.

### The Dark Triad and Unethical and Antisocial Attitudes

Machiavellianism is defined by four facets: distrust in others, desire for control, desire for status, and amoral manipulation (Dahling et al., 2009). This is reflected in research that indicates that Machiavellian workers are more prone to use amoral manipulation, desire power, desire status, distrust others, and engage in unethical, pro-organizational (Castille et al., 2018), and counterproductive work behavior (O'Boyle et al., 2012; Rehman & Shahnawaz, 2018). Kish-Gephart et al. (2010) provided meta-analytic evidence that Machiavellianism promotes unethical choices, which consist of unethical intentions and unethical behaviors. For example, Machiavellianism was positively correlated with motivation to commit fraud, which in turn correlated with a willingness to rationalize effects of fraudulent intention (Harrison et al., 2018). In the Polish sample, Machiavellian attributes resulted in a higher probability of perpetrating bullying, and this tendency increased in workplaces with a hierarchical organizational culture (Pilch & Turska, 2015). Gürlek (2021) identified the positive role of Machiavellianism in unethical behavior intention, both directly and indirectly, through career ambition. In another study (Kuo & Chang, 2021), malicious envy mediated the relationship between Machiavellianism and deviant workplace behavior, while benign envy mediated the relationship between Machiavellianism and organizational citizenship behavior. The findings have contributed to a new understanding of workplace envy’s mediating role in Machiavellianism-driven work behavior.

Machiavellianism can activate in times of organizational change. Employees who scored high in an assessment of Machiavellian beliefs about an organizational change decreased work engagement, which in turn increased turnover intentions (Belschak et al., 2020).

The role of Machiavellian leadership in an organizational context was also tested. Machiavellian leaders have a tendency toward abusive supervision (De Hoogh et al., 2021). In Belschak et al. (2018), Machiavellianism among research employees in combination with leader Machiavellianism explained both stress at work and level of trust in a leader. Links between employees’ Machiavellianism and stress, as with links between employees’ Machiavellianism and trust in a leader, were positively statistically significant for the employees with the highest levels of leader Machiavellianism. In another study, in a group of employees possessing a high level of Machiavellianism, moral reasoning negatively correlated with authentic leadership behavior, but among employees with a low level of Machiavellianism, this relationship was positive. Also, the link between authentic leadership behavior and moral actions was weakened by Machiavellianism (Sendjaya et al., 2016).

In some studies, the influence of narcissism on aggressive behaviors was considered. Youth with narcissistic traits have a tendency toward delinquency, overt aggression, relational aggression, and behavioral and emotional dysregulation (Lau & Marsee, 2013). Among adults, independent of age, sex, and cultural background, narcissism was related to violence and different forms of aggressive behavior, whether indirect, direct, displaced, physical, verbal, or bullying (Kjærvik & Bushman, 2021). A meta-analysis by Du et al. (2022) has shown that the relationship between narcissism and aggression has a universal character. In their study, overall narcissism correlated positively with global, proactive, and reactive forms of aggression.

In many studies, narcissism has been taken into consideration as a predictor of moral intentions and behaviors. In a sample of 63 incarcerated offenders in two prisons, narcissistic personality disorder was positively related to moral disengagement (Brugués et al., 2021). Harrison et al. (2018) showed that narcissism is an antecedent of the motivation to commit fraud, which, indirectly, through a willingness to rationalize, leads to fraudulent intention.

Additionally, numerous studies have indicated the negative function of narcissistic supervisors on their ethical behaviors and the well-being of their subordinates. For example, among leaders and their subordinates in China, narcissism strengthened the negative indirect effect of power distance on leader integrity via higher moral disengagement (Shen et al., 2021). In a study by Scooter and Rogio (2018), a narcissistic CEO proved more prone to fraud misconduct, sexual misconduct, excessive risk-taking misconduct, and general unethical misconduct. Among US employees, the quality of the relationship between a leader and a subordinate was mediated in the link between leader narcissism and both subordinate job satisfaction and emotional exhaustion (Bernerth, 2020). In the same vein, the quality of the relationship between a leader and a subordinate was a mediator in the relationship between leader narcissism and his or her job satisfaction, and perceived social worth. Similarly, Gauglitz et al. (2023) found that narcissistic rivalry among leaders is related to perceived self-esteem threats, which in turn predicts abusive supervision.

Also, psychopathy carries negative consequences for ethical intentions and behaviors like moral disengagement (Brugués et al., 2021; Petruccelli et al., 2017;), bullying (Boddy, 2014; Thakkar et al., 2020; Valentine et al., 2018), fraud intention (Harrison et al., 2018), moral judgment (Marshal et al., 2018), unethical misconduct (Van Scotter & De Déa Roglio, 2020), and counterproductive work behavior (Boddy, 2014). For instance, Harrison et al. (2018) found that psychopathy was indirectly related to fraud intention through a willingness to rationalize. In another study, psychopathic CEOs were more willing to commit fraud misconduct, sexual misconduct, excessive risk-taking misconduct, and general unethical misconduct (Van Scotter & De Déa Roglio, 2020). A longitudinal study conducted on school-attending youth in India revealed that psychopathic traits predicted bullying behavior (Thakkar et al., 2020).

The most exhaustive current research on the topic of psychopathy in prosocial and antisocial attitude contexts was conducted by Sharpe et al. (2023). Three traits of psychopathy—boldness, disinhibition, and meanness—correlated positively with interpersonal deviance, risky behavior, antisocial behavior, psychical and non-physical violence, psychical violence, physical aggression, and rule-breaking. Additionally, disinhibition and meanness were negatively related to prosocialness, emotion recognition, empathy, and social conformity.

Previous studies have emphasized the positive role of dark personality traits in egoism, as measured by the Egoism Scale (Weigel et al., 1999). Studies have confirmed the positive links between egoism and immorality (de Vries & van Kampen, 2010), Machiavellianism (de Vries & van Kampen, 2010; Moshagen et al., 2018), psychopathy (de Vries & van Kampen, 2010; Moshagen et al., 2018), narcissism, sadism, impulsivity, aggression, spitefulness (Moshagen et al., 2018), and pretentiousness (de Vries & van Kampen, 2010). Also, in Raine and Uh (2019) study dark-triad personality traits were positively related to egoism measured by The Selfishness Questionnaire, and additionally, egoism predicted negative psychological outcomes like anxiety and depression.

H5: In a sample of Polish employees, dark-triad personality traits will be positively correlated with an egoistic attitude at work.

### The Dark Triad and Unethical and Antisocial Attitudes: The Moderating Role of an Instrumental Ethical Climate

In the relationships between dark-triad personality traits and attitude at work, some moderators have already been examined, such as abusive supervision (Greenbaum et al., 2018), job autonomy (Rehman & Shahnawaz, 2018), risk of punishment (Jones & Mueller, 2021), leader personality trait (Belschak et al., 2018), and ethical climate (De Hoogh et al., 2021).

Hoogh et al. (2021) showed that the relationship between leader Machiavellianism and abusive supervision was strengthened by an instrumental ethical climate and weakened by a rules ethical climate.

An ethical organizational climate is a contextual factor that can modify the impact of personality traits on attitude at work. The relevant role of different kinds of ethical organizational climates in the link between employees’ emotions and unethical behaviors in the workplace has been noted in various studies. In a sample of Taiwanese employees, a rules ethical climate weakened the positive relationship between negative affect and workplace deviance, but an instrumental ethical climate strengthened this link (Chen et al., 2012). In a similar vein, Wang Xiao (2021) found that an instrumental climate had a moderating effect on the relationship between negative emotions and customer- and coworker-directed deviant behavior, and a caring climate moderated the relationship between negative emotion and organization-, supervisor-, and coworker-directed deviant behavior.

There is a lack of research regarding the relationship between the dark triad and an egoistic attitude at work, as well as the role in this link of an instrumental ethical climate. The interactive function of personality traits and organizational background in explaining employees’ attitude at work is emphasized in Trait-Activation Theory (Tett & Burnett, 2003). Trait-Activation Theory stresses that traits have a tendency to express themselves in work behavior as responses to trait-relevant situational cues perceived as demands, which can be task-oriented, social, or organizational **(**Tett & Burnett, 2003). One of those important organizational cues is an ethical climate reflecting the perception of a moral atmosphere in the work environment and the level of ethics practiced within a company and indicating desirable behaviors in the workplace. The ethical climate is an impactful and significant factor that activates traits that are the base for behavior that is consistent with it, which means that an egoistic attitude at work can express itself as a manifestation of a dark-triad personality, especially in an instrumental ethical climate.

H6: In a sample of Polish employees, an instrumental ethical climate will moderate the relationship between dark-triad personality traits and egoism at work.

To sum up the above, moderated mediation is expected with dark personality traits as mediators between religious practices and egoism at work.

Recent research has indicated an indirect mechanism underlying the relationship between religiosity and unethical behavior intentions and the role of personality traits, such as Machiavellianism, in this connection (Chen & Tang, 2013; Tang & Tang, 2010).

In a longitudinal study among students, intrinsic religiosity was negatively and indirectly related to unethical behavior intentions through Machiavellianism (Tang & Tang, 2010). These results were replicated in Chen and Tang’s (2013) research. Also, Wnuk (2024) showed that humility as a moral virtue mediated the relationship between prayer and egoism at work but only in a group of employees with higher-than-average results in assessing perception of a relationship with God. A negative direct effect of prayer on egoism at work was found only among employees with an average or above-average perception of a bond with God.

H7: In a sample of Polish employees, the indirect effects of religious practices on egoism at work via dark personality traits will be moderated by a perception of a relationship with God and an instrumental ethical climate.

## Methods

### Participants

This study involved 434 full-time employees with employment contracts at different companies in Poland. The survey was anonymous. The following inclusion criteria to study were applied: adulthood (at least 18 years old) and current employment, regardless of the type of contract. The questionnaires were available online and encompassed statements regarding the aim of the study, its anonymous and voluntary nature, and the possibility of withdrawing from the study at any time without any consequences. Every participant expressed consent to participate in the study. Participants recruit respondents using social media (mainly via Facebook and LinkedIn) and subsequent respondents were obtained using the snowball method. The sample was balanced in terms of gender and education. The sociodemographic characteristics of the study sample are presented in Table 1.

**Table 1:** Sociodemographic characteristics of the study sample (N = 434)

Variables	N	%
*Gender*		
Men	179	41.2
Women	255	58.8
Age (years; M ± SD)	29.44 (± 10.79)	
Seniority	8.66 years (± 9.48)	
*Educational level*		
Elementary	6	1.4
Vocational	7	1.6
Secondary	182	41.9
Higher	239	55.1
*Level the position held*		
Ordinary workers	190	43.8
Independent specialist	148	34.1
Low managers	30	6.9
Middle managers	51	11.8
Senior managers	15	3.5

### Measures

#### Perception of Relationship with God

The participants’ relationship with God was examined through one subscale of an Employee Spirituality Scale. (Wnuk, 2022b). A short version of this measure was chosen, which consisted of six following items: In difficult moments at work I turn to my Higher Power (for example, God); I ask my Higher Power (for example, God) for help in doing my daily duties at work; The Higher Power (for example, God) gives me hope that matters at work will move in the right direction; My Higher Power (for example, God) is a source of comfort for me at work; I am sure that my Higher Power (for example, God) will help me manage in difficult moments at work; Thanks to the Higher Power (for example, God) I am able to overcome my limitations at work.

For each item, participants answered on a five-point Likert scale (from strongly agree = 5 to strongly disagree = 1). Confirmatory factor analysis (CFA) confirmed the model goodness of the six-item solution (χ^2^[4] = 1.96; *p* = 0.743; CMIN/df = 0.49; CFI = 1; NFI = 0.99; TLI = 1; RMSEA = 0.000 (90% CI [0.000. 0.052]); SRMR = 0.002), explaining 89.66% of the variance of a relationship with a “Higher Power” (God).

### Prayer

On the frequency of prayer scale, participants responded to one question that addressed the frequency of prayer, choosing one from the following five possibilities: never (1), sometimes (2), once monthly (3), once weekly (4), and every day (5).

#### Mass Attendance

Mass attendance was examined on the basis of a five-point scale consisting of (1) never, with the exception of baptisms, weddings, or funerals, (2) a few times a year, (3) 1 or 2 times monthly, (4) 2 or 3 times monthly, and (5) once per week or more.

#### Dark-Triad Traits

The dark triad was measured by the Polish version of the Dirty Dozen Scale (Czarna et al., 2016). The three-factor structure of this tool—encompassing narcissism, Machiavellianism, and psychopathy—was confirmed. Each factor consisted of four items, answered on a five-point Likert scale (from strongly agree = 5 to strongly disagree = 1). This measure had good internal consistency and test–retest reliability. In this study, the three-factor structure of the Dirty Dozen Scale was confirmed as having a good fit to the data (χ^2^[40] = 65.77; *p* = 0.006; CMIN/df = 1.64; CFI = 0.98; NFI = 0.96; TLI = 0.97; RMSEA = 0.039 (90% CI [0.021. 0.055]); SRMR = 0.036) that explained 62.78% of the variance of the dark triad.

#### Instrumental Ethical Climate

The presence of an instrumental work climate was measured using items from the Polish version Ethical Work Climate Questionnaire (EWCQ) (Wnuk et al., 2025) that are indicators of an instrumental type of ethical climate. This tool verifies employees’ perceptions of an ethical work climate and categorizes it into the following types: instrumental, caring, law and code, rules, and independence (Victor & Cullen, 1988; Wnuk et al., 2025). The instrumental ethical climate in the Polish version of EWCQ encompasses four questions.

Items are rated on a five-point Likert scale (from 1 = strongly disagree to 5 = strongly agree). The instrumental type of climate demonstrated a satisfactory internal consistency reliability coefficient, with a Cronbach’s alpha of 0.71 (Victor & Cullen, 1988). The CFA confirmed a good fitness to the data for items constituting the instrumental organizational climate (*χ*^2^[8] = 30.21; *p* = 0.000; CMIN/df = 3.77; CFI = 0.96; NFI = 0.95; TLI = 0.91; RMSEA = 0.080 (90% CI [0.051. 0.111]); SRMR = 0.046), explaining 51.14% of the variance of this construct.

#### Egoism at Work

Egoism at work was examined using the Egoism at Work Attitude Questionnaire (EWAQ) prepared by Wnuk (2024). It is a one-dimensional measure containing eight items rated on a five-point Likert scale (from strongly agree = 5 to strongly disagree = 1). The results of CFA confirmed that the one-dimensional solution was a good fit to the data (χ^2^[11] = 7.9; *p* = 0.722; CMIN/df = 0.718; CFI = 1; NFI = 0.99; TLI = 1; RMSEA = 0.000 (90% CI [0.000. 0.029]); SRMR = 0.007), explaining 51.32% of the variance of this construct.

### Conceptual Model and Statistical Analysis

**Fig. 1:**
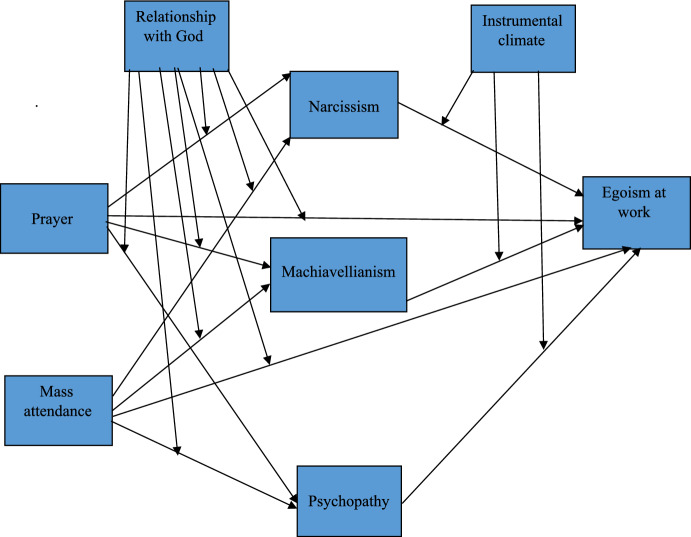
Conceptual model

The model presented in Fig. 1 covers moderated mediation with religious practices (prayer and Mass attendance) as independent variables, a perception of a relationship with God as a moderator in the link between religious practices and dark-triad personality traits, and egoism at work as a dependent variable, as well as an instrumental ethical climate as a moderator in the link between the dark triad and egoism at work. The following controlled variables were applied: education, sex, seniority, level of position held, and age.

It was expected that religious practices were negatively related to the dark triad and egoism in the workplace, but only in a group of employees with a perception of a strong relationship with God, and that, in turn, the instrumental ethical climate strengthened the positive relationships between the dark-triad traits and egoism at work.

For the statistical analyses, IBM SPSS AMOS version 26.0 was used. Model number 22 of the template for PROCESS for SPSS prepared by Hayes (2018) was used. The bootstrapping method was applied based on 5000 resamples within a 95% bias-corrected confidence interval. Model number 22 serves to verify moderated mediation with two different moderators; one moderator regards the relationship between the independent variable and the potential mediator, and the second references the relationship between the potential mediator and the dependent variable.

If the moderation effect within the path analysis was identified, the sample was divided into three groups, with analyses of moderation with probe interactions on –1 SD, mean, and + 1 SD, reflecting “low,” “medium,” and “high” values of that variable (see Aiken & West, 1991). Additionally, the Johnson–Neyman output was used (Hayes, 2018) to examine the relationship between the independent variable and the dependent variable for regions of significance across levels of the moderator variable.

The variance inflation factor (VIF) was employed to verify potential multicollinearity problems (Witten et al., 2011). Common method bias was examined by Harman’s single factor method in SPSS and a common latent factor in AMOS (Podsakoff et al., 2003).

## Results

### Preliminary Results

In Table 2, descriptive statistics are presented. Due to the fact that the values of the VIF indicator were lower than the threshold (Witten et al., 2011), multicollinearity was not found.

**Table 2:** Descriptive statistics (N = 434)

Variable	Minimum	Maximum	Mean	Standard deviation	Skewness	Kurtosis	IF	Cronbach’s alpha coefficient
Prayer	1	5	2.1	1.54	1.1	-.46	4.5	-
Mass attendance	1	5	2	1.49	1.19	-.19	3.7	-
Relationship with God	6	30	12.69	7.33	.82	-.52	2.85	.98
Narcissism	4	20	11.33	3.37	-.01	-.26	1.3	.77
Machiavellianism	4	20	8.05	3.22	.08	.62	1.85	.82
Psychopathy	4	20	8.67	3.07	.74	.47	1.68	.75
Instrumental climate	7	34	19.31	4.33	.11	.01	1.15	.76
Egoism at work	8	37	17	5.77	.66	.34		.86

VIF = variance inflation factor

The values of the Pearson coefficients (*r*) are shown in Table 3. Prayer and Mass attendance correlated positively with a perception of a relationship with God, seniority, education, level of position held, and age, and related negatively to narcissism, psychopathy, and egoism at work. A relationship with God was positively correlated with seniority, education, level of the position held, and age and negatively related to egoism at work. Narcissism was positively related to Machiavellianism, psychopathy, an instrumental ethical climate, egoism at work, and level of position held and negatively correlated with seniority and age. Machiavellianism was positively correlated with psychopathy, egoism at work, and an instrumental ethical climate and was negatively related to seniority and age. Psychopathy was positively related to egoism at work and an instrumental ethical climate and negatively correlated with education, seniority, and age.

**Table 3:** Values of r-Pearson correlation coefficients between research variables (N = 434)

	1	2	3	4	5	6	7	8	9	10	11
1. Prayer											
2. Mass attendance	.84**										
3. Relationship with God	.79**	.74**									
4. Narcissism	− .13**	− .17**	− .06								
5. Machiavellianism	− .03	− .08	.01	.45**							
6. Psychopathy	− .17**	-.17**	. − 08	.29**	.60**						
7. Instrumental climate	− .04	− .06	.02	.13**	.3**	.33**					
8. Egoism at work	− .22**	− .24**	− .14**	.32**	.55**	.61**	.45**				
9. Education	.22**	.22**	.19**	.03	− .05	− .11*	− .02	− .12*			
10. Seniority	.45**	.19**	.38**	− .18**	− .15**	− .12*	− .01	− 21**	.30**		
11. Level of the position held	.18**	.20**	.17**	.11**	− .01	− .04	− .02	− .15**	.31**	.48**	
12. Age	.44**	.43**	.38**	− .21**	− .18**	− .15**	− .02	− .24**	.37**	.95**	.48**

^*^p < .05, **p < .01,

The one-factor solution explained 25.27% of the variance (acceptable threshold 40%) (Podsakoff et al., 2003). Also, the differences between standardized regression weights for every item in a model with six latent factors compared to a model with one latent factor were lower than 0.2. It suggested the absence of the potential common method bias problem.

### Hypotheses Testing

A lack of relevant relationships between prayer and narcissism (*b* = −0.14, SE = 0.18, 95% CI [−0.515, 0.221]), psychopathy (*b* = −0.15, SE = 0.17, 95% CI [−0.491, 0.189]), and Machiavellianism (*b* = 0.24, SE = 0.19, 95% CI [−0.128, 0.614]), as with Mass attendance and both psychopathy (*b* = −0.13, SE = 0.17, 95% CI [−0.473, 0.203]) and Machiavellianism (*b* = −0.1, SE = 0.18, 95% CI [−0.465, 0.268])—but not narcissism (*b* = −0.47, SE = 0.18, 95% CI [−0.837, −0.111])—only partially supported the hypothesis 1. Only one religious practice, Mass attendance, was negatively related to one dark-triad personality trait, narcissism.

Statistically significant interactive effects of prayer and a perception of a relationship with God on narcissism, psychopathy, and Machiavellianism, as well as interactive effects of Mass attendance and a relationship with God on psychopathy, but not Machiavellianism, and narcissism, were noticed (see Table 4), providing partial support for hypothesis 2. Interactive effects of prayer and a perception of a relationship with God explained an additional 1% of variance of narcissism, 3.8% of variance of psychopathy, and 2.3% of variance of Machiavellianism. Also, interactive effects of Mass attendance and a perception of a relationship with God explained an additional 2.1% of variance of psychopathy. These moderating effects (Aiken & West, 1991) are presented in Tables 5 and 6. Negative relationships between prayer and both narcissism and psychopathy (see Table 5), as well as between Mass attendance and psychopathy (see Table 6), were found only in a group of employees with higher-than-average results in the assessment of their relationship with God. Also, prayer was positively related to Machiavellianism only among employees with lower-than-average outcomes regarding a perception of a relationship with God (see Table 5).

**Table 4:** Results of moderation analyses (N = 434)

Hypotheses	Moderating variable	Predictor	Coefficient	t	p	LLCI	ULCI
H2 (outcome: narcissism)	Relationship with God	Prayer	− .038	− 2.20	.024	− .071	− .004
H2 (outcome: Machiavellianism)			− .052	− 3.60	.002	− .085	− .018
H2 (outcome: psychopathy)			− .066	− 4.20	.000	− .096	− .035
H4 (outcome: egoism at work)			.042	1.91	.056	− .001	.085
H2 (outcome: narcissism)		Mass attendance	− .009	− .57	.569	− .043	.240
H2 ( outcome: Machiavellianism)			− .027	− 1.53	.124	− .060	007
H2 (outcome: psychopathy)			− .049	− 3.07	.002	− .080	− .017
H4 (outcome: egoism at work)			.056	2.58	.010	.013	.099
H6 (outcome: egoism at work)	Instrumental climate	Narcissism	.016	1.13	.259	− .011	.043
		Psychopathy	.062	3.20	.001	.024	.099
		Machiavellianism	− .038	− 2.16	.031	− .072	− .003

LLCI = 95% confidence interval (low); ULCI = 95% confidence interval (high)

**Table 5:** Moderating effects of prayer on dark-triad dimensions for different values of relationship with God as moderator (N = 434)

Relationship with God	Effect	Outcome variable	*t*	*p*	LLCI	ULCI
− 6.63 (− 1 *SD*)	.105	narcissism	.41	.686	− .406	.617
.00 (*M*)	− .148		− .79	.430	− .519	.221
7.31 (+ 1 *SD*)	− .429		− 2.50	.012	− .766	− .091
− 6.63 (− 1 *SD*)	.59	Machiavellianism	2.25	.024	.076	1.103
.00 (*M*)	.243		1.28	.198	− .128	.614
7.31 (+ 1 *SD*)	− .148		− .80	.421	− .476	.199
− 6.63 (− 1 *SD*)	.286	psychopathy	1.19	.232	− .184	.756
.00 (*M*)	− .151		− .87	.384	− .491	.189
7.31 (+ 1 *SD*)	− .632		− 4.01	.000	− .942	− .322

LLCI = 95% confidence interval (low); ULCI = 95% confidence interval (high)

**Table 6:** Moderating effects of mass attendance on psychopathy and egoism at work for different values of relationship with God as moderator (N = 434)

Relationship with God	Effect	Outcome variable	*t*	*p*	LLCI	ULCI
-6.63 (− 1 *SD*)	.191	psychopathy	.77	.438	− .293	.695
.00 (*M*)	− .135		.78	.432	− .519	.221
7.31 (+ 1 *SD*)	− .495		− 3.38	. .000	− .766	− .091
− 6.63 (− 1 *SD*)	− .951	egoism at work	− 2.81	.005	− 1.616	− .284
.00 (*M*)	− .576		− 2.42	.015	− 1.043	− .109
7.31 (+ 1 *SD*)	− .163		− ..79	.424	− .564	.238

LLCI = 95% confidence interval (low); ULCI = 95% confidence interval (high)

Hypothesis 3, which was about negative relationships between religious practices and egoism at work, was partially supported. Prayer was not statistically significantly related to egoism at work (b = −0.44, SE = 0.24, 95% CI [−0.911, 0.035]), but Mass attendance was (*b* = −0.58, SE = 0.23, 95% CI [−1.143, −0.109]). Also anticipated in hypothesis 4, the interactive effects of religious practices and a perception of a relationship with God on egoism at work was partially confirmed. Moderation was noted only in reference to Mass attendance but not prayer (see Table 6). The interactive effects of religious practices and a perception of a relationship with God explained an additional 0.68% of variance of egoism at work. Only in those employees who scored average in a perception of a relationship with God and lower-than-average in Mass attendance was negatively related to egoism at work. Unexpectedly, a perception of a relationship with God weakened the connection between Mass attendance and egoism at work (Table 6).

Consistent with hypothesis 5, narcissism, psychopathy, and Machiavellianism were positively related to egoism at work, respectively: (*b* = 0.18, SE = 0.06, 95% CI [0.047, 0.313]), (*b* = 0.59, SE = 0.06, 95% CI [0.423, 0.759]), and (*b* = 0.33, SE = 0.08, 95% CI [0.178, 0.498]). Hypothesis 6, which was about the interactive effects of dark-triad personality traits and an instrumental ethical climate on egoism at work, was partially supported. An instrumental ethical climate moderated the link between psychopathy as well as Machiavellianism and egoism at work but not between narcissism and egoism at work (see Table 4). The interactive effects of psychopathy and an instrumental ethical climate explained an additional 1% of variance of egoism at work, and the interactive effects of Machiavellianism and an instrumental ethical climate explained an additional 0.4% of variance of egoism at work. In three groups of employees with low, average, and high results in the assessment of instrumental ethical climate, psychopathy was positively related to egoism at work (Table 7). Higher outcomes in the category of instrumental ethical climate were connected with a higher positive correlation between psychopathy and egoism at work. An inverse effect was noticed with respect to the relationship between Machiavellianism and egoism at work. An instrumental ethical climate weakened this positive link. Positive correlations between Machiavellianism and egoism at work were found only in employees with average and lower-than-average outcomes in the category of instrumental ethical climate, but in the second group, the strength of this link was bigger (Table 7).

**Table 7:** Moderating effects of dark-triad traits on egoism at work for different values of instrumental organizational climate as moderator (N = 434)

Instrumental climate	Effect	Predictor	*t*	*p*	LLCI	ULCI
− 4.35 (− 1 *SD*)	.494	Machiavellianism	4.25	.000	.265	.722
.00 (*M*)	.338		4.16	.000	.1787	.498
4.35 (+ 1 *SD*)	.192		1.69	.091	− .028	.394
− 4.35 (− 1 *SD*)	.328	psychopathy	2.43	.015	.062	.593
.00 (*M*)	.591		6.93	.000	.423	.759
4.35 (+ 1 *SD*)	.855		8.07	.000	.646	1.063

LLCI = 95% confidence interval (low); ULCI = 95% confidence interval (high)

Hypothesis 7, regarding the indirect effects of religious practices on egoism at work via dark-triad personality traits moderated by a perception of a relationship with God and an instrumental ethical climate, was partially confirmed. Double moderated mediation was noticed only for the effects of prayer on egoism at work through psychopathy (−0.004, SE = 0.001, 95% CI [−0.007, −0.001]) and Machiavellianism (0.002, SE = 0.001, 95% CI [0.001, 0.004]) as well as Mass attendance on egoism at work through psychopathy (−0.003, SE = 0.001, 95% CI [−0.006, −0.001]). There was no double moderated mediation for the effects of prayer on egoism at work and Mass attendance on egoism at work through narcissism, respectively (−0.001, SE = 0.001, 95% CI [−0.002, 0.001]), and (−0.001, SE = 0.001, 95% CI [−0.001, 0.001]). Also double moderated mediation for the effect of Mass attendance on egoism at work through Machiavellianism was irrelevant (0.001, SE = 0.001, 95% CI [−0.001, 0.003]).

The indicator of conditional moderated mediation by instrumental ethical climate for the relationship between prayer and egoism at work and Machiavellianism as mediator was statistically significant but only for the average and less-than-average results of the instrumental ethical climate, respectively (−0.002, SE = 0.001, 95% CI [−0.037, −0.005]) and (−0.003, SE = 0.001, 95% CI [−0.055, −0.007]).

## Discussion

The purpose of this study was to examine the mechanisms underpinning the connection between religious practices and egoism in the workplace and the role of dark-triad personality traits as mediators, as well as a relationship with God and an instrumental ethical climate as moderators in this link.

First of all, achieved results showed that frequency of prayer and Mass attendance as behavioral manifestations of employee religious devotion are not sufficient to prevent dark-triad personality traits, except narcissism, and that they require a close relationship with God as a form of bonding religious expression (Saroglou, 2011). These findings are consistent with the assumption of relational spirituality (Davis et al., 2021) and previous research conducted on this topic, which emphasized that the role of prayer in promoting moral virtues, such as humility, is conditional and depends on a perception of relationship with God (Jankowski et al., 2022; Krause & Hayward, 2014; Wnuk, 2024). A perception of bond with God is significant in this process, being a source of support, direction, stress relief, and transcendence and helping with the overcoming of barriers and obstacles in the workplace (Wnuk, 2022b). What is important is that religion as a cognitive schema (McIntosh, 1995) in socialization processes can provide meaning, purpose, values, rules, habits, and ways of acting, which, through internalization, shapes desirable and valuable traits and virtues and prevents negative ones, such as narcissism, psychopathy, and Machiavellianism.

Perception of relationship with God strengthened the negative link between prayer and Machiavellianism as well as between prayer and Mass attendance with psychopathy. These results could be explained by the beneficial function of religious socialization, which balances the impact of parental rearing on the development of Machiavellianism and psychopathy (Jonason et al., 2014; Yendell et al., 2022), and within the religious practices allows building a close relationship with God and thanks to that can modify and limits manifestations of cynicism, self-centeredness, manipulation, and exploitation of others.

It is worth noticing that Mass attendance was independent of the perception of relationship with God and negatively related to narcissism, but in reference to prayer, this negative link was strengthened by a perception of relationship with God. This means that both forms of religious practices can serve as a way to tame and mitigate narcissism. Roman Catholic religious doctrine stresses humility and not self-aggrandizement as a desirable virtue and models a servant attitude based on the figure of Jesus Christ as a full exemplification of this way of life. The obtained results confirmed that the effect of protection from narcissism is enhanced during prayer by the perception of a stronger relationship with God. This can be explained by the confrontation in a prayer act, where mortal, finite, fleeting, and fragile men turn to infinite, immortal, and omniscient God and experience this asymmetrical relationship, forcing them to admit their ontological and epistemological limitations. Narcissism was taken into account without the division into two forms of narcissism: overt and covert. Both of them differ in attitude to God and praying. A covert narcissist needs God to protect him or her as a vulnerable individual, but an overt narcissist avoids God as a source of threat to his or her grandiosity and power over others (Zondag & Uden, 2014).

In contrast with previous research, a relationship with God did not moderate the link between prayer and egoism at work (Wnuk, 2024). It is important to note that the moderating effect was close to a statistically significant level (0.056), and the division of the employees into the three groups, with a lower-than-average, average, and higher-than-average level of perception of relationship with God, showed that a relationship with God strengthened the negative link between prayer and egoism at work. Interestingly, perceived a bond with God weakened the negative link between Mass attendance and egoism at work. The above relationship was weaker for the group of employees with an average or lower-than-average score in the perception of a bond with God category, and it was not statistically sound among employees with the perception of the strongest relationship with God. This implies that Mass attendance is an efficient way to protect against egoism at work, but more frequent use of this practice does not increase this effect but leads to a decrease. One possible explanation of this interactive effect of Mass attendance and a perception of a relationship with God on egoism at work is that among employees with a strong relationship with God, Mass attendance fulfills another function than reducing egoistic motivation in the workplace. The religious approach relies on enhancing positive and prosocial manifestations of faith but also focuses on preventing and challenging antisocial manifestations.

The appropriate framework to interpret these results is Catholic Church teachings, according to which the essence of being a real Christian is altruism, and egoism, as an opposite phenomenon, is a source of sin and inequity. The best exemplification of this and the model to be imitated is Jesus Christ, who came to serve; in his activities, there were no selfish motivations. Also, it is possible that Mass attendance is not an intrinsic but an extrinsic religious motivation manifestation, and building a relationship with God through church attendance is not the way to limit antisocial behavior at work but fulfills other goals. Following Allport (1967) intrinsic religiosity based on religion as a central and autotelic value and motivational force in life is related to positive outcomes, but extrinsic religiosity that treats religion as an instrumental value and a means to achieve non-religious aims can lead to negative effects (Błażek & Besta, 2012; Vitell et al., 2009). This means that mass attendance can be externally motivated and focused not on religious growth but on other secular purposes, such as searching for support or filling the need for affiliation.

According to expectations, dark-triad personality traits of employees positively predicted their egoistic motivation in the workplace. Additionally, an ethical instrumental climate amplified the positive link between psychopathy and egoism at work but surprisingly weakened the link between Machiavellianism and egoism. In all dark-triad traits, the common element is self-centeredness (Kay & Arrow, 2022), which, in connection with other characteristics of those personality profiles can lead to an egoistic approach in the workplace and motivates exploitative, manipulative, and callous behavior (Jones & Figueredo, 2013).

Inconsistent with trait activation theory (Tett & Burnett, 2003), situational and environmental factors, such as an ethical instrumental climate consistent with the dark-triad traits, did not strengthen a positive connection between narcissism and egoism at work and unexpectedly decreased the relationship between Machiavellianism and egoism. This “latent potential,” as described by Tett and Burnett (2003), that can anticipate workplace behavior was activated by an ethical instrumental climate facilitating the expression individuals of egoistic motivation as consistent with the psychopathy trait.

In this case, an ethical instrumental climate provided cues that triggered the individual predispositions of psychopathic employees to behave egoistically in the workplace but not narcissistic individuals. Probably narcissistic employees did not perceive an instrumental ethical climate as an opportunity for egoistic behavior in the workplace, but among those possessing a Machiavellian inclination, this factor discouraged egoism at work. As was shown, both psychopaths and Machiavellian employees were sensitive to environmental factors, such as an instrumental ethical climate, in relation to egoism at work, but in different ways. These discrepancies can be explained by the differences between these character traits and the specificity of their expression. Previous studies focused on Machiavellianism as important to business ethics (Jones & Mueller, 2021), mainly due to its susceptibility to external environmental influences (De Hoogh et al., 2021), in contrast to psychopathy, which exhibits a stable and consistent set of behaviors independent of contextual factors (Hoppenbrouwers et al., 2015; Jones & Neria, 2019). The studies emphasized two key differences between them: impulsivity control and sensitivity to punishment (Jones & Mueller, 2021). First of all, in this study, an ethical climate was an incentive to behave egoistically, and this kind of attitude in the workplace was rewarded rather than punished. Secondly, the Machiavellian characteristics of flexibility and impulsivity control are lacking in psychopathic individuals.

According to the achieved results, the instrumental ethical climate was a potential trigger for psychopaths to act in an egoistic way but unexpectedly an inhibitor of egoistic motivation in Machiavellians. Employees with psychopathic traits were not forced to delay gratification or inhibit behavioral responses and planning (Patton et al., 1995) because their behavior was coherent with environmental requirements and gave them a chance to fulfill their needs without having to overcome the lack of these skills. In contrast to psychopathy, using advantages attributable to Machiavellianism in achieving goals like adapting behavior to contextual demands (Bereczkei, 2015) and applying more sophisticated forms of manipulation was not necessary to attain egoistic goals because the organizational environment was congruent with Machiavellian characteristics. Employees did not need to put additional effort into realizing egoistic plans, and stronger encouragement of egoistic attitudes contained in an instrumental ethical climate paradoxically led to lower egoism at work. Due to the transparency of expectations from the perception of an instrumental ethical climate consistent with Machiavellian motivations, employees did not need to take veiled forms of action to hide their real intentions. That is probably why the increased expression of Machiavellian attributes in a stronger instrumental climate weakened their egoistic motivation.

These findings are contrary to the previous approach to psychopathy as a trait insensitive to contextual external factors due to problems with impulse control, invulnerability to punishment, and a lack of adaptive resources to react to environmental demands, which lead to predictable, consistent behavior (Jones & Mueller, 2021). They indicated that psychopathy can be a stronger positive predictor of egoistic motivation in the workplace if the cues and incentives that come from the perception of an ethical instrumental climate are stronger. On the other hand, this congruency between psychopathic characteristics and external organizational environmental requirements is not proof of flexibility. We do not know whether egoism at work for psychopathically inclined employees would be affected when, according to the concept of trait activation, the ethical climate is inconsistent with psychopathic “latent predispositions.” Only future research on psychopathic behavior in other types of ethical climates could potentially answer this question precisely.

Also contrary to earlier research, an unexpected direction to the interactive effect of Machiavellianism and an instrumental ethical climate on egoism at work was observed. Previous studies unequivocally stated that this type of ethical climate activates Machiavellian traits and strengthens, not weakens, their impact on unethical behavior. In accordance with these findings, the flexibility of Machiavellian personality traits as a factor sensitive to organizational context was confirmed.

The moderating effect of a perception of a relationship with God between prayer and psychopathy and also Mass attendance and psychopathy was additional support that this character trait can be susceptible to change due to external factors, and the perception of a bond with God is an important influence on this link.

The mediating role of Machiavellianism in the relationship between religious practices and egoism at work was supported, but only in reference to prayer and the average and less-than-average level of instrumental ethical climate.

### Research Implications

A plethora of theoretical implications can be derived from the achieved results. First of all, the mechanisms underlying the relationship of religious practices with dark-triad personality traits and egoism at work differ from each other, with one exception—psychopathy as a mediator. According to the findings, both prayer and Mass attendance play a protective role against narcissism, psychopathy, and egoism at work, and additionally, prayer in interaction with a perception of a relationship with God prevents Machiavellianism. The indirect effects of Mass attendance on egoism at work through narcissism were independent of a perception of a relationship with God and an ethical instrumental climate. The mechanism underpinning the link between prayer and egoism at work, with Machiavellianism’s mediating role, was relevant only for employees who scored average and less than average regarding an instrumental ethical climate.

What should be emphasized, contrary to previous conclusions, is that psychopathy was the trait that was susceptible to the influence of environmental factors, which implies that individuals with psychopathic traits can take into consideration the contextual framework of their behavior and adapt to external organizational demands. Also, contrary to previous studies, an instrumental climate didn’t strengthen but weakened the positive effect of Machiavellianism on egoism.

From a practical point of view, the most important outcome of this research would be raising awareness of the significance of religious practices among organizational representatives, leaders, managers, and employees responsible for human resources, especially the significance of religious practices as protection against the expression of antisocial personality traits and egoistic motivation in the workplace.

Encouraging stakeholders to create an ethical climate would strengthen this effect. Different educational methods like training, workshops, or e-learning can serve as a way to realize this goal. Managers should have knowledge about the impact of religious involvement on business ethics to build an organizational environment inclusive of these practices and tolerant of different manifestations of spirituality. According to the concept of trait activation (Tett & Burnett, 2003), CEOs and other individuals responsible for organizations should be aware of the influence of an ethical instrumental climate in revealing Machiavellian and psychopathic personality traits that lead to egoistic behaviors at work. One way to protect against unethical behavior in the workplace is to shape organizational culture to focus on dimensions contrary to instrumental ethical climate, such as femininity, cooperativeness, and the need for affiliation. It would also help to reward the expression of different forms of prosocial behavior. From a recruitment perspective, another option for avoiding dark-triad traits is the implementation by recruitment specialists and HR representatives of appropriate recruitment methods to identify the dark-triad personality traits as undesirable to candidates through interviews, assessment centers, etc. In doing this, recruiters should be aware that candidates with these traits will not be interested in revealing them to employers and that, using the manipulative skills they possess, they will present themselves as desirable candidates. This means that, for example, psychometrical tests or other recruitment tools based on declaration will not be adequate to check for these traits.

### Limitations and Future Research

The conducted study has some limitations. First, the research outcomes' generalizability is limited to Polish Roman Catholic employees. Due to the fact that denomination and national level of religiosity can moderate the link between religiosity and both personality traits and attitude toward work, future research on this topic should be conducted in more religiously and culturally diverse settings, in more secular nations, and among representatives of religious affiliations other than Roman Catholic. Previous research has shown that cultural aspects like levels of religiosity and welfare on the national level are moderators of the relationship between religiosity and some outcomes, like life satisfaction (Diener et al., 2011; Lun & Bond, 2013).

To achieve a more comprehensive picture of this topic, other aspects of religion should be taken into account—not only in terms of the behavioral dimensions of religion but also believing and belonging (Saroglou, 2011)—to examine which aspect is most important in counteraction of antisocial traits and behavior. Understanding which aspects of religion are the most beneficial would be important for the practical implementation of methods that encourage employees toward these forms of religious devotion.

To explore and better understand how prayer potentially protects against the expression of negative personality traits and antisocial behavior in the workplace, it is recommended that different types of prayer be used, such as adoration, thanksgiving, reception, confession, and supplication (Laird et al., 2009).

Additionally, in contrast to egoism motivation, the use of measures regarding altruism manifestations in the workplace could have interesting results, showing whether the identified mechanism is universal and can be generalized to prosocial attitudes at work or whether it is reserved only for egoism. In future research, besides the dark-triad personality traits as mediators between religiosity and egoism at work, some desirable virtues like gratitude, forgiveness, or compassion should be added. An earlier study has confirmed the positive role of prayer in shaping humility, which in turn negatively predicted egoism at work (Wnuk, 2024).

The next step in this topic should be examining whether other types of organizational ethical climates interact with personality traits in explaining egoistic attitudes in the workplace. Consistent with trait activation theory (Tett & Burnett, 2003), personality traits like those of the dark triad should be inhibited in a climate that is not favorable to selfishness, such as a caring climate where other behaviors, focused on cooperativeness, empathy, and compassion, are required. Also, not general narcissism but the trifurcated concept of narcissism with all its manifestations should be examined, as it may lead to potentially different consequences in the workplace (Schneider et al., 2023).

Finally, a longitudinal, not cross-sectional, study could confirm identified mechanisms from a cause-and-effect perspective. Applying a cross-sectional design does not guarantee that the presented direction of the variables is correct, especially since alternative models were not verified, and so the design does not exclude, for example, the possibility that religious practices are antecedents of egoism at work, which in turn is positively related to dark-triad personality traits.

## Conclusion

This study aimed to examine the mechanisms underlying prayer and egoism at work, which assume that the negative link between religious practices and dark-triad personality traits is strengthened by a perception of a positive relationship with God and, in turn, dark-triad personality traits are positively linked with egoism at work, and instrumental ethical climate amplify this link. As was confirmed in the sample of Polish employees perceived a bond with God strengths the protecting function of prayer from dark-triad traits, and stronger perception of instrumental ethical climate enhances the negative effect of psychopathy on egoism at work and unexpectedly dampens the negative effect of Machiavellianism on egoism at work. The preventive role of both prayer and Mass attendance from egoism at work was found due to the benevolent image of God, which limited psychopathy in employees using these religious practices.

## Declarations

### Human and Animal Participations

All procedures performed in studies involving human participants were in accordance with the ethical standards of the institutional and/or national research committee and with the 1964 Helsinki declaration and its later amendments or comparable ethical standards. Every participant expressed written consent to take part in the study.
